# The relationship of peritubular capillary density with glomerular volume and kidney function in living kidney donors

**DOI:** 10.1007/s40620-023-01734-5

**Published:** 2023-09-28

**Authors:** J. van der Weijden, P. A. De Hoogt, M. M. E. Leufkens, A. A. Keijbeck, H. van Goor, M. C. van den Heuvel, J. P. M. Cleutjens, C. Moers, M. G. Snoeijs, G. J. Navis, M. van Londen, I. M. Nolte, S. P. Berger, M. H. De Borst, C. J. Peutz-Kootstra

**Affiliations:** 1 Division of Nephrology, Department of Internal Medicine, University Medical Center Groningen, University of Groningen, Hanzeplein 1, P.O. Box AA53, 9713 GZ Groningen, The Netherlands; 2 Department of Vascular Surgery, Maastricht University Medical Center+, Maastricht, The Netherlands; 3 Department of Pathology, Maastricht University Medical Center+, Cardiovascular Research Institute Maastricht (CARIM), Maastricht, The Netherlands; 4 Department of Pathology and Medical Biology, University Medical Center Groningen, University of Groningen, Groningen, The Netherlands; 5 Department of Surgery, University Medical Center Groningen, University of Groningen, Groningen, The Netherlands; 6 Department of Epidemiology, University Medical Center Groningen, University of Groningen, Groningen, The Netherlands; 7 Department of Pathology, Gelre Ziekenhuizen, Apeldoorn, The Netherlands

**Keywords:** Living kidney donation, Kidney biopsy, Peritubular capillary rarefaction, Glomerular hypertrophy, Kidney function

## Abstract

**Background:**

Peritubular capillary rarefaction plays an important role in the progression of chronic kidney disease. Little is known about the relation between peritubular capillary density, glomerular volume and filtration rate in the healthy kidney.

**Methods:**

In this single-center study, we included 69 living kidney donors who donated between 2005 and 2008 and had representative renal biopsies available. In all donors, glomerular filtration rate was measured using ^125^I-Iothalamate before donation and at five years after donation. Before donation, the increase in glomerular filtration rate after dopamine stimulation was measured. Glomerular volume and peritubular capillary density were determined in biopsies taken at the time of transplantation. Pearson’s correlation coefficient and linear regression were used to assess relations between parameters.

**Results:**

Mean donor age was 52 ± 11 years and mean measured glomerular filtration rate was 119 ± 22 mL/min before donation and 82 ± 15 mL/min at five years after donation. While peritubular capillary density (measured by either number of peritubular capillaries/50,000 μm^2^ or number of peritubular capillaries/tubule) was not associated with measured glomerular filtration rate before or after donation, number of peritubular capillaries/tubule was associated with the increase in measured glomerular filtration rate after dopamine stimulation (St.*β* = 0.33, *p* = 0.004), and correlated positively with glomerular volume (*R* = 0.24, *p* = 0.047). Glomerular volume was associated with unstimulated measured glomerular filtration rate before donation (St.*β* = 0.31, *p* = 0.01) and at five years (St.*β* = 0.30, *p* = 0.01) after donation, independent of age.

**Conclusions:**

In summary, peritubular capillary density was not related to unstimulated kidney function before or after kidney donation, in contrast to glomerular volume. However, number of peritubular capillaries/tubule correlated with the increase in glomerular filtration rate after dopamine stimulation in healthy kidneys, and with glomerular volume. These findings suggest that peritubular capillary density and glomerular volume differentially affect kidney function in healthy living kidney donors.

**Graphical abstract:**

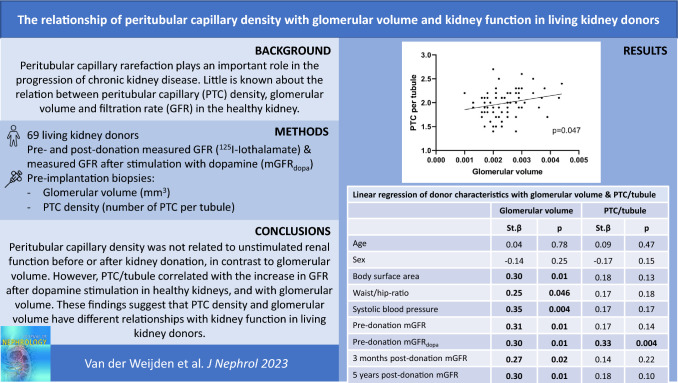

## Introduction

Microstructural changes such as glomerular hypertrophy, interstitial fibrosis and tubular atrophy can be present to various degrees in kidneys of healthy individuals without clinical signs of kidney damage [1]. Glomerular volume is positively associated with single-nephron glomerular filtration rate (GFR) in healthy individuals, probably as a compensation mechanism to maintain a normal total GFR in the case of loss of nephrons or increased renal demand [2]. Moreover, a higher glomerular volume is associated with hypertension, overweight, height and family history of end-stage kidney disease [2, 3]. Glomerular enlargement has been explained as the result of either increased intraglomerular pressure or an increased glomerular ultrafiltration coefficient, accompanied by prolongation of glomerular capillaries and subsequent enlargement of the glomerular tuft [4, 5]. Indeed, hypertrophic glomeruli have more capillaries, and a greater total capillary area [6, 7]. It is unknown whether these glomerular capillary changes also affect the peritubular capillaries (PTCs), and if so, whether PTC density is also related to kidney function in the healthy kidney.

The peritubular capillary bed predominantly evolves from the efferent glomerular arteriole [8, 9], while the glomerular capillary bed is situated behind the afferent arteriole. A single nephron unit consists of a glomerulus with accompanying tubular system, in which distal tubuli “return” to their own glomerulus, but the PTC microcirculation forms a coalescing plexus surrounding tubuli from different nephrons. Both cortical capillary beds are highly permeable to water and solutes which are filtered in the glomerulus and almost totally reabsorbed via tubuli in peritubular capillaries. They differ in blood pressure as well as in oxygen tension: blood pressure and oxygen levels are high in the glomerulus, while blood pressure is lower and there is a steep decrease in oxygen gradient in the interstitium [9, 10]. In patients with insulin-dependent diabetes mellitus, an independent relationship of glomerular and interstitial biopsy parameters with renal function was found [11]. Based on these differences between the glomerular and peritubular capillary beds we hypothesize that an increase in glomerular volume is not accompanied by an increase in peritubular capillaries in healthy kidneys. We expect that in early stages of kidney damage, a phase of glomerular capillary hypertrophy occurs followed by peritubular capillary loss and fibrosis in later stages of chronic kidney disease (CKD).

An ideal setting to study microstructural parameters as glomerular volume and PTC density in healthy kidneys is in living kidney donors, for whom pre-implantation biopsies are often available. Previous kidney biopsy studies in living kidney donors showed that glomerular hypertrophy is associated with higher pre-donation GFR [2], but with lower short- and long-term post-donation GFR [12, 13]. It also has been shown that a higher body mass index (BMI) was associated with glomerular hypertrophy [14], and a reduced increase in GFR in response to a dopamine stress test [15]. Thus, in this study, we investigated the relation between PTC density and glomerular volume, pre- and post-donation-measured-GFR in a cohort of living kidney donors.

## Methods

### Study population

For this retrospective cohort study, we identified 73 living kidney donors with representative kidney biopsies. Biopsies were taken right after donor nephrectomy (T1), right before implantation (T2) and/or after reperfusion (T3) and were considered representative if T1, T2 and/or T3 had a total cortical surface of minimally 0.6 mm^2^ with at least 5 glomeruli. All donors donated between August 11, 2005 and June 17, 2008 at the University Medical Center Groningen, The Netherlands. Four donors were excluded because they were part of the Dutch “cross-over” program and only came to our center for the actual nephrectomy procedure, rendering 69 living kidney donors eligible for inclusion in this study. All donors underwent pre- and three-month post-donation clinical and laboratory measurements as part of the regular living kidney donor screening program. In 52 donors, five-year post-donation follow-up was available. In 2014, these data were added to the TransplantLines Biobank and Cohort study (ClinicalTrials.gov identifier: NCT03272841). This is an observational cohort study on short- and long-term outcomes after organ transplantation/donation, as described previously [16]. The study was approved by the institutional ethical review board (METc 2014/077). All procedures were conducted in accordance with the declaration of Helsinki and declaration of Istanbul.

### Biopsy analysis

**Fig. 1:**
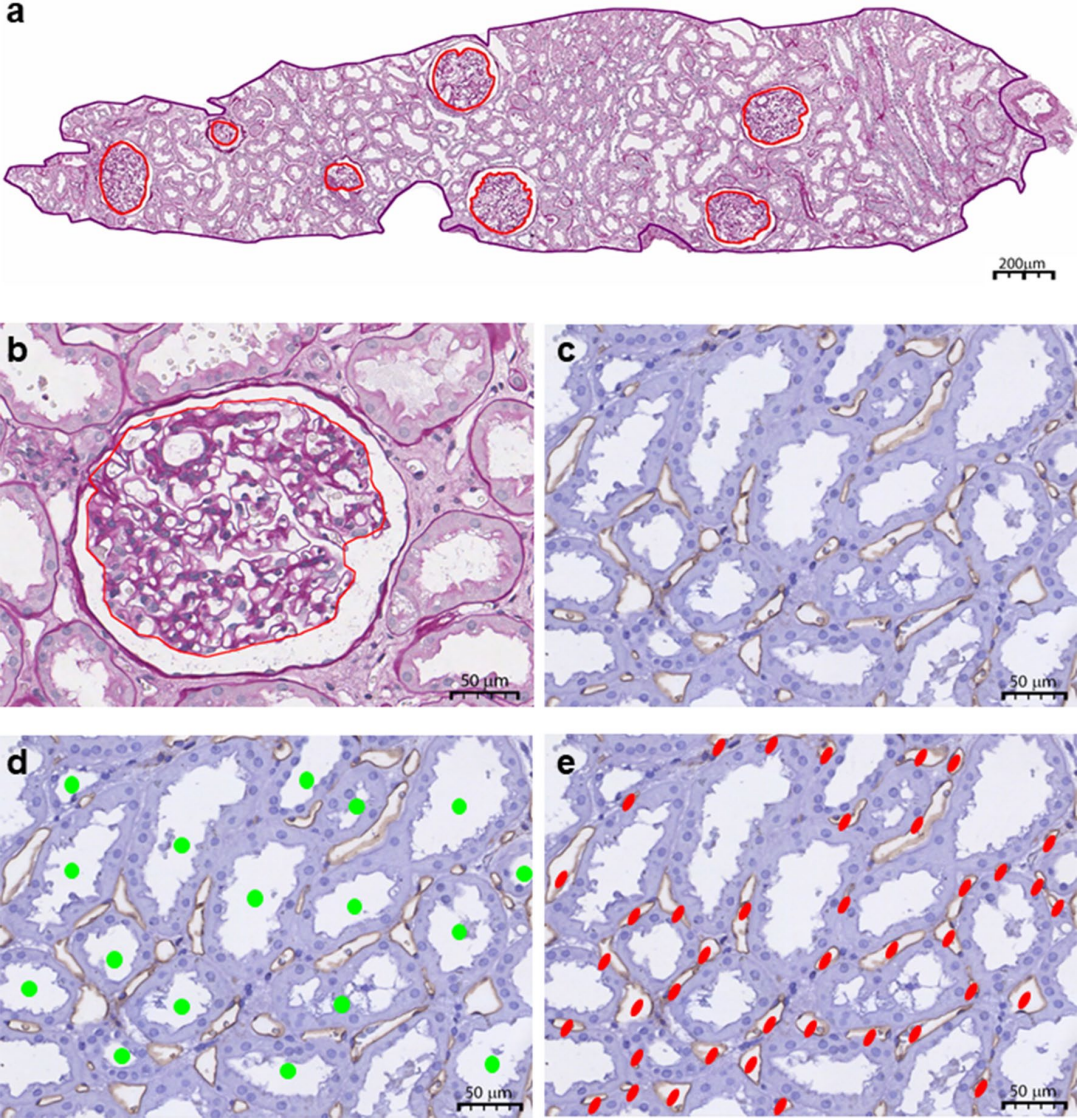
Representative examples of the microstructural measurements on biopsies. In Periodic acid-Schiff (PAS) stained sections (**a** and **b**) the area of cortex was delineated (**a**), and the area of the tuft of all individual non-sclerosed glomeruli (**a** and **b**). On CD34 stained sections (**c**–**e**) peritubular capillaries (PTCs) are accentuated. Tubuli (**d**) peritubular capillaries (**e**), were annotated manually. Scale bars: **a** 200 um; **b**–**e** 50 um

All available T1, T2 and T3 biopsies were stained with periodic-acid-shiff (PAS) and, on a separate section, an immunohistochemical staining for CD34 (Monosan, Uden, the Netherlands) was performed. In brief, parafin-embedded tissue sections were incubated with primary antibody after blocking of endogeneous perioxidase and antigen retrieval by boiling in TRIS EDTA buffer. After washing, the biopsies were incubated with bright vision anti-mouse HRP (Immunologic; Duiven, The Netherlands) followed by washing and thereafter 3,3-diaminobenzidine (DAB) (DAKO cytomation, Glosturp, Denmark) was used as the chromogen. Thereafter the protocol slides were counterstained with hematoxylin (Klinipath, Duiven, The Netherlands). Periodic-acid-shiff and immunohistochemically stained slides were digitalized using a Ventana scanner (Ventana iScan HT (Roche, Basel, Switzerland), and imported in Panoramic Image Viewer (3DHistotech, Budapest, Hungary); examples are shown in Fig. 1. Microstructural parameters were measured on PAS-stained sections by one observer (ML), according to Elsherbiny et al. [14], with the exception that partial glomeruli were counted as 1 and not as 0.5. Briefly, total cortical biopsy area was annotated manually, as well as glomerular tuft surface area of all non-sclerotic glomeruli. Then the profile area of non sclerotic glomeruli was calculated by dividing the number of non sclerotic glomeruli by cortical area. The Weibel Gomez stereological model was used to calculate the non sclerotic glomeruli density. Furthermore, non sclerotic glomeruli volume (glomerular volume) was calculated as described by Elsherbiny et al. [14]. Of all CD34 stained sections a maximum of 10 pictures of 120,000 μm^2^ were taken in a serpentine manner [17], with Panoramic Viewer 1.15.4 and exported as jpeg into Paint (Microsoft, Seattle, WA, USA). There were no glomeruli present in these pictures. In all pictures, PTCs and tubules were manually traced by one observer (ML), with the exclusion of interlobular arteries. Peritubular capillaries and Tubuli per picture were quantified by Image J. Peritubular capillary density was assessed as number of PTCs per tubule (PTC/tubule) and number of PTCs per surface area (PTC/50,000 μm^2^).The tubular area was determined by dividing the area of the pictures with the number of tubuli counted per biopsy.

In cases that met our inclusion criteria of at least 5 glomeruli and 600,000 μm^2^ of cortex, the PAS stained digital section was scored histologically according to Banff by a pathologist (CPK) [18]. Grade of interstitial fibrosis and tubular atrophy (IF/TA) was determined as highest of tubular atrophy (ct) or interstitial fibrosis (ci). Also, IF/TA was assessed by the pathologist as more or less than 5% of the cortical area.

### Assessment of kidney function and other clinical measurements

During screening, clinical parameters as weight, height, hip circumference, waist circumference and blood pressure were measured, medication use was asked as well as smoking history. Kidney function before and at three months and five years after donation was indirectly determined by measuring the clearance of the exogenous filtration marker ^125^I-iothalamate (measured GFR (mGFR), described in more detail previously) [19]. In short, ^125^I-Iothalamate and ^131^I-hippurate infusions were started and after a stabilization period, baseline measurements were performed in a steady state of plasma tracer levels. Clearances were calculated as (U*V)/P and (I*V)/P, where U*V represents the urinary excretion, I*V represents the infusion rate of the tracer and P represents the plasma tracer concentration per clearance period. We calculated mGFR from clearance levels of these tracers using (U*V)/P and corrected the renal clearance of ^125^I-iothalamate for urine collection errors by multiplying the urinary ^125^I-Iothalamate clearances with the ratio of plasma and urinary ^131^I-hippurate clearance by using the following formula:

$${\text{Corrected}}\,{\text{Clearance}}_{{{\text{iot}}}} = \frac{{{\text{Clearance}}_{{{\text{hip}}}} \left( {I \times V/P} \right)}}{{{\text{Clearance}}_{{{\text{hip}}}} \left( {U \times V/P} \right)}} \times {\text{Clearance}}_{{{\text{iot}}}} \left( {U \times V/P} \right)$$

The mGFR after stimulation with dopamine was also assessed before donation (mGFR_dopa_). The mGFR_dopa_ was used to calculate the dopamine-induced increase in GFR (ΔmGFR_dopa_, previously referred to as the renal functional reserve (RFR) [19, 20]) by subtracting the unstimulated mGFR form the mGFR_dopa_. Dopamine-stimulated mGFR was missing in 4 cases. Serum creatinine was measured routinely in our central chemistry laboratory by an isotope dilution mass spectrometry (IDMS) traceable enzymatic assay on the Roche Modular (Roche Ltd., Mannheim, Germany). In addition, serum HbA1c concentration was recorded.

### Statistical analyses and sample size estimation

Data are reported as mean (standard deviation (SD)) for normally distributed variables and median [interquartile range, IQR] for skewed data. Binary variables are shown as “number (%)”. Correlations between glomerular volume, IF/TA, PTC/tubule, tubular area and PTC/50,000 µm^2^ were assessed by scatter plots and Pearson’s correlation coefficients. In cross-sectional analyses, we investigated which pre-donation characteristics were associated with the microstructural parameters using univariable linear regression analyses. Subsequently, we used linear regression analyses to assess the association between the morphometrical parameters and pre- and post-donation kidney function outcomes. Outcomes were pre- and three months and five year post-donation mGFR. All univariable associations of the microstructural parameters with pre- and post-donation outcomes were adjusted for age using multivariable linear regression analyses, because age is a known determinant of GFR, as well as microstructural features in the kidney [2, 21]. To detect a correlation of 0.3 with an *α* of 0.05 and a power of 80%, 67 donors are needed. Statistical analyses were performed in SPSS version 28 for Windows (IBM, Armonk, NY), and Graphpad Prism 8 for Windows (Graphpad, San Diego, CA). *p* values of < 0.05 were considered statistically significant.

## Results

### Pre- and post-donation characteristics

A total of 69 living kidney donors were included in this study. Mean age was 52 ± 11 years, 46% were female and all donors were white (Table 1). The donors had a mean BMI of 26 ± 4 kg/m^2^ and a mean systolic blood pressure (SBP) of 130 ± 15 mmHg. Three donors had a pre-donation serum HbA1c level ≥ 6.5%, of which two donors had a BMI of 34 and 35 kg/m^2^, respectively. Pre-donation mGFR was 119 ± 22 mL/min and decreased to 75 ± 14 at three months post-donation (Table 2). Five years after donation, mGFR was 82 ± 15 mL/min. Before donation, mean glomerular volume was 0.0024 ± 0.0007 mm^3^, mean number of PTC/tub was 1.97 ± 0.3, mean number of PTC/50,000 μm^2^ was 25.9 ± 4.4, mean tubular area was 3679.2 ± 835.7 µm^2^, and 19 donors had > 5% IF/TA (Table 3).

**Table 1:** Baseline characteristics of the living kidney donor population

Variable	Pre-donation
*N*	69
Age, years	52 ± 11
Sex, *N* (%} female	33 (46)
Race, *N* (%) white	69 (100)
Weight, kg	81 ± 13
Length, cm	176 ± 8
BMI, kg/m^2^	26 ± 4
BSA, m^2^	1.97 ± 0.17
Hip size, cm	97 ± 7
Waist size, cm	92 ± 9
Waist/hip-ratio	0.95 ± 0.08
SBP, mmHg	130 ± 15
DBP, mmHg	77 ± 9
Serum HbA1c, %	5.7 ± 0.8
Serum creatinine, mmol/L	79 ± 13
Smoking, *N* (%) smokers	23 (32)

Characteristics at donor evaluation

*BMI* body mass index, *BSA* body surface area, *SBP* systolic blood pressure, *DBP* diastolic blood pressure, *N* number

**Table 2:** Characteristics of the pre- and post-donation kidney function parameters

Variable	Before donation	3 months post-donation	5 years post-donation
*N*	69	69	53
mGFR, mL/min			
Mean ± SD	119 ± 22	75 ± 14	82 ± 15
Range	85–209	50–112	51–119
mGFR_dopa_, mL/min			
Mean ± SD	127 ± 20	75 ± 13	n.a.
Range	90–175	50–122	
ΔmGFR_dopa_, mL/min*			
Mean ± SD	9 ± 7	n.a.	n.a.
Range			

One donor had a pre-donation mGFR of 209, in this donor dopamine-stimulated mGFR was not available. After this donor, the highest pre-donation mGFR was 159

*mGFR* measured glomerular filtration rate, _*dopa*_ under stimulation of dopamine, *n.a.* not applicable, *N* number, *SD* standard deviation

*Calculated as mGFR_dopa_ – mGFR

**Table 3:** Microstructural characteristics of the donor kidneys

Variable	
*N*	69
Number of non-sclerotic glomeruli (*n*)	17.1 ± 8.6
Cortical area (mm^2^)	6.0 ± 2.6
NSG area, (µm^2^)	20,879 ± 10,266
Glomerular volume (mm^3^)	0.0024 ± 0.0007
Glom area density (glomeruli/mm^2^)	2.92 ± 0.92
Glomerular density (glomeruli/mm^3^)	19.68 ± 7.49
Profile tubular area, µm^2^	3679.2 ± 835.7
PTC/tubule	1.97 ± 0.3
PTC/50,000 µm^2^	25.9 ± 4.4
Any tubular atrophy	58 (73%)
IF/TA > 5%	19 (24%)

Measured in pre-implantation biopsies of the transplanted donor kidney

*N* number, *NSG* non-sclerotic glomeruli, *PTC* peritubular capillaries, *IF/TA* interstitital fibrosis and tubular atrophy

### Correlations between microstructural parameters

**Fig. 2:**
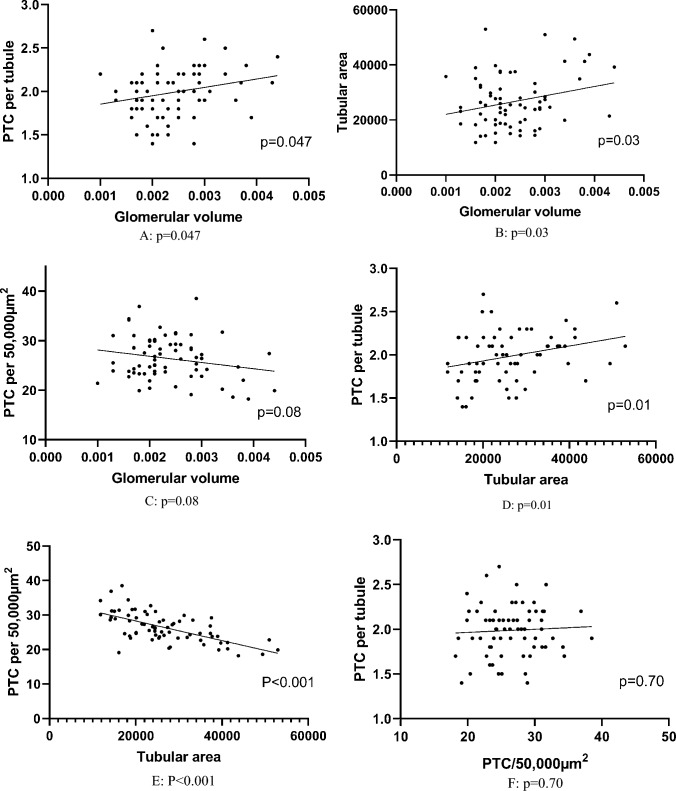
Scatter plots of the morphometrical parameters with each other. **A** glomerular volume (*x*-axis) with PTC/tubule (*y*-axis), Pearson = 0.24, *p* = 0.047; **B** glomerular volume (*x*-axis) with tubular area (*y*-axis), Pearson = 0.26, *p* = 0.03; **C** glomerular volume (*x*-axis) with PTC/50,000 µm^2^ (*y*-axis), Pearson = − 0.21, *p* = 0.08; **D** tubular area (*y*-axis) with PTC/tubule (*x*-axis), Pearson = 0.31, *p* = 0.01; **E** tubular area (*y*-axis) with PTC/50,000 µm^2^ (*x*-axis), Pearson = 0.63, *P* < 0.001; **F** PTC/50,000 µm^2^ (*y*-axis) with PTC/tubule (*x*-axis), Pearson = 0.05, *p* = 0.70. PTC/tubule: peritubular capillary per tubule; PTC/50,000 µm^2^: peritubular capillary per 50,000 µm^2^

Scatterplots of correlations between microstructural parameters are shown in Fig. 2. The strongest correlation was observed for tubular area with PTC/50,000 μm^2^ (*R* = − 0.63, *p* < 0.001), with fewer PTCs per 50,000 μm^2^ in cases with larger tubular area. However, when the number of PTCs was adjusted for the number of tubules on the biopsy (PTC/tubule), we observed an increase in PTC/tubule in cases with increased tubular area (*R* = 0.31, *p* = 0.01), which is as expected because cases with larger tubules display a smaller number of tubules per surface area on the biopsy. Glomerular volume correlated positively with tubular area (*R* = 0.26, *p* = 0.03) and with PTC/tub (*R* = 0.24, *p* = 0.047), and negatively with a trend towards significance with PTC/50,000 μm^2^ (*R* = − 0.21, *p* = 0.08). There was no correlation between PTC/tubule and PTC/50,000 μm^2^ (*R* = 0.05, *p* = 0.70).

### Clinical determinants of microstructural parameters in living donor kidney biopsies

Univariable linear regression analyses did not reveal associations of clinical variables (e.g. age, sex, weight, blood pressure) with PTC/tubule or PTC/50,000 μm^2^ (Table 4). Body surface area (BSA) (St.*β* = 0.30, *p* = 0.01), waist/hip-ratio (St.*β* = 0.25, *p* = 0.05), systolic blood pressure (SBP, St.*β* = 0.35, *p* = 0.004) and diastolic blood pressure (DBP, St.*β* = 0.30, *p* = 0.01) were all positively associated with glomerular volume (Table 4). A trend towards significance was shown for the association of BMI with glomerular volume (St.*β* = 0.23, *p* = 0.06). Smoking correlated negatively and significantly with tubular area (St.*β* = − 0.38, *p* = 0.004). Living kidney donors with IF/TA > 5% in their biopsy were older than donors without IF/TA (*t*-test *p* = 0.002, Table 5). Also, individuals with IF/TA > 5% had a larger tubular area (Table 5). None of the clinical parameters were associated with PTC/tubule or PTC/50,000 μm^2^.

**Table 4:** Univariable linear regression analysis of pre-donation variables with morphometrical parameters

	PTC/tubule		PTC/50,000 µm^2^		Glomerular volume		Tubular area	
	St.*β*	*p*	St.*β*	*p*	St.*β*	*p*	St.*β*	*p*
Age	0.09	0.47	− 0.15	0.23	0.04	0.78	0.18	0.16
Sex	− 0.17	0.15	0.05	0.67	− 0.14	0.25	− 0.13	0.29
BMI	0.06	0.64	− 0.03	0.83	0.23	0.06^b^	− 0.09	0.46
BSA	0.18	0.13	− 0.09	0.48	0.30	0.01^a^	0.10	0.43
Waist/hip-ratio	0.17	0.18	− 0.02	0.88	0.25	0.047^a^	− 0.11	0.42
SBP	0.17	0.17	− 0.17	0.19	0.35	0.004^a^	0.21	0.09
DBP	0.01	0.99	− 0.17	0.17	0.30	0.01^a^	0.10	0.43
Serum HbA1c	0.03	0.85	0.25	0.06	0.13	0.33	− 0.13	0.34
Serum creatinine	− 0.04	0.72	0.06	0.62	− 0.07	0.55	− 0.06	0.63
Smoking	− 0.17	0.21	0.20	0.14	− 0.09	0.50	− 0.38	0.004^a^

Univariable linear regression analysis showing associations of donor characteristics at donor evaluation with the microstructural parameters measured in pre-implantation biopsies

*BMI* body mass index, *BSA* body surface area, *DBP* diastolic blood pressure, *PTC/tubule* peritubular capillary per tubule, *PTC/50,000µm*^*2*^ peritubular capillary per 50,000 µm^2^, *SBP* systolic blood pressure

^a^*p* < 0.05

^b^*p* < 0.10

**Table 5:** Characteristics of the population according to IF/TA percentage

Variable	IF/TA > 5%	IF/TA < 5%	*p* value
*N*	15	54	–
Age, years	59 ± 8	49 ± 10	0.002^a^
Sex, *N* (%} female	5 (33)	26 (48)	0.31
Race, *N* (%) Caucasian	16 (100)	56 (100)	–
BMI, kg/m^2^	27 ± 5	26 ± 3	0.37
BSA, m^2^	1.96 ± 0.20	1.97 ± 0.16	0.86
Waist/hip-ratio	0.99 ± 0.10	0.94 ± 0.07	0.08^b^
SBP, mmHg	134 ± 16	129 ± 15	0.24
Serum HbA1c, %	5.9 ± 1.2	5.6 ± 0.5	0.16
Serum creatinine, mmol/L	76 ± 15	80 ± 12	0.35
Smoking, *N* (%) smokers	6 (40)	17 (31)	0.54
mGFR, mL/min	119 ± 21	119 ± 22	0.87
mGFR_dopa_, mL/min	126 ± 22	127 ± 20	0.88
ΔmGFR_dopa_, mL/min	8 ± 5	10 ± 7	0.35
eGFR, mL/min/1.73m^2^	89 ± 10	87 ± 14	0.76
Glomerular volume	0.0027 ± 0.0009	0.0023 ± 0.0006	0.09^b^
PTC/tub	2.1 ± 0.3	2.0 ± 0.3	0.15
Tubular area	312,278 ± 10,354	25,104 ± 8893	0.01^a^
PTC/50,000 m^2^	25 ± 4	27 ± 4	0.20

Data presented as mean ± standard deviation. Differences between characteristics were tested using an independent *T*-test for normally distributed continuous variables and with a *χ*^2^ test for proportional variables

*BMI* body mass index, *BSA* body surface area, *SBP* systolic blood pressure, *DBP* diastolic blood pressure, *mGFR* measured glomerular filtration rate, _*dopa*_ under stimulation of dopamine, *eGFR* estimated glomerular filtration rate, *IF/TA* interstitial fibrosis and tubular atrophy, *N* number, *PTC/tubule* peritubular capillary per tubule, *PTC/50,000* *µm*^*2*^ peritubular capillary per 50,000 µm^2^

^a^*p* < 0.05

^b^*p* < 0.10

### Associations of microstructural parameters with pre-donation GFR

Peritubular capillary/tubule was significantly and independent of age associated with the ΔmGFR_dopa_ (= dopamine induced increase in mGFR, St.*β* = 0.25, *p* = 0.04, Table 6), but not with unstimulated mGFR (St.*β* = 0.17, *p* = 0.14). Peritubular capillary/50,000 µm^2^ was not associated with mGFR or ΔmGFR_dopa_ (St.*β* = 0.01, *p* = 0.97 and St.*β* = 0.04, *p* = 0.74, respectively). Glomerular volume was significantly and positively associated with pre-donation mGFR (St.*β* = 0.31, *p* = 0.01, Table 6), but not with the ΔmGFR_dopa_ (St.*β* = − 0.13, *p* = 0.31). Tubular area and IF/TA were not associated with pre-donation kidney function (Table 6). In a multivariable linear regression model including glomerular volume and PTC/tubule, both were independently associated with pre-donation mGFR_dopa_ (*R*^2^ = 0.29), with glomerular volume being associated with pre-donation mGFR, and PTC/tub with pre-donation ΔmGFR_dopa_ (Table 7). The association of PTC/tubule with mGFR_dopa_ and ΔmGFR_dopa_ remained significant after adjustment for tubular area (PTC/tubule with mGFR_dopa_: St.*β* = 0.29, *p *= 0.01; PTC/tubule with ΔmGFR_dopa_: St.*β* = 0.26, *p* = 0.045, Table 8).

**Table 6:** Association of microstructural parameters with pre-donation kidney function

Independent variable	Outcome	St.*β*	*p*	*R*^2^
PTC/tubule	Pre-donation mGFR	0.17	0.14	0.15
	Pre-donation mGFR_dopa_	0.33	0.004^a^	0.23
	Pre-donation ΔmGFR_dopa_	0.25	0.04^a^	0.06
PTC/50,000 µm^2^	Pre-donation mGFR	0.01	0.97	0.12
	Pre-donation mGFR_dopa_	0.00	0.99	0.13
	Pre-donation ΔmGFR_dopa_	0.04	0.74	− 0.004
Glomerular volume	Pre-donation mGFR	0.31	0.01^a^	0.22
	Pre-donation mGFR_dopa_	0.30	0.01^a^	0.22
	Pre-donation ΔmGFR_dopa_	− 0.13	0.31	0.01
Tubular area	Pre-donation mGFR	0.14	0.24	0.14
	Pre-donation mGFR_dopa_	0.21	0.08^b^	0.17
	Pre-donation ΔmGFR_dopa_	0.04	0.79	− 0.01
IF/TA	Pre-donation mGFR	− 0.11	0.35	0.13
	Pre-donation mGFR_dopa_	− 0.10	0.43	0.13
	Pre-donation ΔmGFR_dopa_	− 0.17	0.19	0.02

Linear regression analysis of the microstructural parameters (measured in pre-implantation biopsies), displayed in the left column, with different pre-donation kidney function outcomes, shown in the second column. Univariable standardized beta, *p* values and *R*^2^ are shown. All analyses adjusted for age, ΔmGFR_dopa_ = GFR_dopa_ – GFR (= dopamine-induced GFR increase, in literature referred to as “renal functional reserve”)

*mGFR* measured glomerular filtration rate, *mGFR*_*dopa*_ measured glomerular filtration rate after stimulation with dopamine, *PTC/tubule* peritubular capillary per tubule, *PTC/50,000* *µm*^*2*^ peritubular capillary per 50,000 µm^2^, *IF/TA* interstitial fibrosis and tubular atrophy

^a^*p* < 0.05

^b^*p* < 0.10

**Table 7:** Multivariable linear regression analysis of glomerular volume and PTC/tubule with pre-donation mGFR, adjusted for age

Independent variables	Outcome	St.*β*	*p*	*R*^2^
Glomerular volume	Pre-donation mGFR	0.29	0.01^a^	0.22
PTC/tubule		0.13	0.26	
Age		− 0.42	< 0.001^a^	
Glomerular volume	Pre-donation mGFR_dopa_	0.25	0.02^a^	0.29
PTC/tubule		0.29	0.01^a^	
Age		− 0.43	< 0.001^a^	
Glomerular volume	Pre-donation ΔmGFR_dopa_	− 0.17	0.17	0.07
PTC/tubule		0.28	0.03^a^	
Age		− 0.17	0.17	

Multivariable models to investigate whether the association between glomerular volume and pre-donation mGFR was independent of PTC/tubule and age, and to investigate whether the association between PTC/tubule and the ΔmGFR_dopa_ was independent of glomerular volume and age

ΔmGFR_dopa_ = GFR_dopa_ – GFR (= renal functional reserve); GV = glomerular volume; PTC/tub = PTC/tubule

Regression equations (age was centered around the mean):

$$predonation\,mGFR=77.8+9105.7\times GV+10.3\times PTC/tub-0.86\times age$$

$${predonation\,mGFR}_{dopa}=67.6+7320.0\times GV+21.7\times PTC/tub-0.83\times age$$

$${predonation\,\Delta mGFR}_{dopa}=-0.8-1683.9\times GV+7.2\times PTC/tub-0.11\times age$$

^a^*p* < 0.05

^b^*p* < 0.10

**Table 8:** Multivariable linear regression analysis of PTC/tubule and tubular area with pre-donation mGFR, adjusted for age

Independent variables	Outcome	St.*β*	*p*	*R*^2^
PTC/tubule	Pre-donation mGFR	0.15	0.22	0.15
Tubular area		0.10	0.39	
Age		− 0.41	< 0.001^a^	
PTC/tubule	Pre-donation mGFR_dopa_	0.29	0.01^a^	0.24
Tubular area		0.14	0.24	
Age		− 0.43	< 0.001^a^	
PTC/tubule	Pre-donation ΔmGFR_dopa_	0.26	0.045^a^	0.04
Tubular area		− 0.03	0.83	
Age		0.18	0.16	

Multivariable models to investigate whether the association between PTC per tubule and the pre-donation ΔmGFR_dopa_ was independent of tubular area and age

ΔmGFR_dopa_ = GFR_dopa_ – GFR (= renal functional reserve); TA = tubular area; PTC/tub = PTC/tubule

Regression equations (age was centered around the mean):

$$predonation\,mGFR=89.5+11.9\times PTC/tub+0.00\times TA-0.85\times age$$

$${predonation\,mGFR}_{dopa}=75.8+22.2\times PTC/tub+0.00\times TA-0.83\times age$$

$${predonation\,\Delta mGFR}_{dopa}=-3.2-6.7\times PTC/tub-0.000020\times TA-0.12\times age$$

^a^*p* < 0.05

^b^*p* < 0.10

### Associations of microstructural parameters with post-donation GFR

There was no association of PTC/tubule with unstimulated mGFR at three months or five years post-donation. Glomerular volume was significantly and positively associated with both three-month, and five-year post-donation mGFR (St.*β* = 0.27, *p* = 0.02 and St.*β* = 0.30, *p* = 0.01, respectively, Table 9). Tubular area, PTC/50,000μm^2^ and IF/TA were not associated with post-donation mGFR (Table 6).

**Table 9:** Association of microstructural parameters with post-donation kidney function

Microstructural parameter	Outcome	St.*β*	*p*	*R*^2^
PTC/tubule	3-month post-donation mGFR	0.14	0.22	0.15
	5-year post-donation mGFR (*n* = 52)	0.18	0.10^b^	0.39
PTC/50,000 µm^2^	3-month post-donation mGFR	0.06	0.64	0.13
	5-year post-donation mGFR (*n* = 52)	− 0.05	0.67	0.35
Glomerular volume	3-month post-donation mGFR	0.27	0.02^a^	0.20
	5-year post-donation mGFR (*n* = 52)	0.30	0.01^a^	0.44
Tubular area	3-month post-donation mGFR	0.14	0.25	0.15
	5-year post-donation mGFR (*n* = 52)	0.08	0.50	0.36
IF/TA	3-month post-donation mGFR	− 0.14	0.26	0.14
	5-year post-donation mGFR (*n* = 52)	0.05	0.64	0.35

Linear regression analysis of the microstructural parameters (measured in pre-implantation biopsies), displayed in the left column, with different post-donation kidney function outcomes, shown in the second column. Univariable standardized beta, *p* values and *R*^2^ are shown. All analyses adjusted for age

*mGFR* measured glomerular filtration rate, *mGFR*_*dopa*_ measured glomerular filtration rate after stimulation with dopamine, *PTC/tubule* peritubular capillary per tubule, *PTC/50,000* *µm*^*2*^ peritubular capillary per 50,000 µm^2^, *IF/TA* interstitial fibrosis and tubular atrophy

^a^*p* < 0.05

^b^*p* < 0.10

## Discussion

The present study aimed to investigate the relationship between peritubular capillary density and other microstructural parameters including glomerular volume, tubular area and IF/TA in healthy kidneys. Furthermore, we investigated whether PTC density and other microstructural parameters were associated with clinical characteristics and pre- and post-donation-measured GFR. In this study we confirm associations of glomerular volume with mGFR, systolic blood pressure and body size measurements at donation. We found no association of PTC density (measured by either PTC/50,000 μm^2^ or PTC/tubule) with clinical characteristics or pre- or post-donation mGFR. However, we did find a positive association between PTC/tubule and ΔmGFR_dopa_. Our results indicate that glomerular volume and peritubular capillary density have a differential relationship with kidney function. In addition, our findings suggest that an increase in glomerular capillaries (i.e. glomerular volume) is not associated with an increase in number of peritubular capillaries in healthy individuals. Peritubular capillary density may therefore not provide prognostic information in potential living kidney donors.

It has been broadly recognized that peritubular capillary rarefaction plays an important role in the development of interstitial fibrosis and tubular atrophy and the progression of CKD [22–25]. In recipients of a kidney from a deceased donor, an average decrease in the PTC/tubule ratio of nearly 25% in the first three months after transplantation is associated with lower graft function [7]. Gaining knowledge on how PTCs react to early compensatory/pathological microstructural changes in the kidney can contribute to better understanding their role in the development of CKD. We observed a negative correlation (with trend towards significance) between PTC/50,000 μm^2^ and glomerular volume, i.e. larger glomerular volume is associated with fewer peritubular capillaries in the pre-implantation biopsy. In a case report of two cases with low birth weight (known to be associated with low nephron number and CKD), proteinuria and polycythemia, a decreased PTC per surface area was also found together with glomerular hypertrophy [26]. The association between glomerular volume and tubular area that we observed was in line with previous findings [14]. The positive relationship of glomerular volume with PTC/tubule that we found is likely due to a combination of a decrease in PTC density and an increase in tubular area (i.e., fewer tubules per picture) in individuals with larger glomeruli. Experimental studies show that even subtle alterations in tubular cells [27] or pericytes [28, 29] can induce PTC loss and IF/TA, indicating that the tubulovascular ratio (measured by PTC/tubule) provides additional information besides counting PTC numbers per surface area.

Even though PTC density is clearly decreased in advanced CKD [22–25], we found no association of PTC density with kidney function in our cohort, possibly because only healthy kidneys with normal GFR were included in this study. Total GFR is the result of single nephron GFR and number of nephrons [2], so it would be interesting for future studies to investigate whether PTC density is in fact related to single-nephron GFR, and whether this explains the lack of an association with total GFR in healthy kidneys. Our finding that IF/TA in the pre-donation biopsy is not related to measured GFR post-donation confirms results from Buus et al. [30]. We observed that individuals with more than 5% IF/TA had an increased tubular area and (a trend towards) a larger glomerular volume. In biopsies of patients with IgA nephropathy and various forms of chronic tubulointerstitial disease, hypertrophic tubuli expressed vascular endothelial growth factor (VEGF), which did not protect from PTC loss with concomitant loss of renal function [31, 32]. Further studies are needed to investigate whether tubular hypertrophy may be a first response to glomerular enlargement in healthy individuals, that, if not compensated for by an increase in PTC density, might lead to decreased tubular oxygen supply resulting in tubular atrophy, PTC loss, interstitial fibrosis, and renal function decline.

While PTC density was not associated with pre- or post-donation mGFR, we did find an association between PTC/tubule and the GFR increase after dopamine infusion (ΔmGFR_dopa_). Dopamine infusion induces dilatation of the afferent and efferent arterioles, and the GFR increase after dopamine infusion has been referred to as “renal stress testing” [19]. As hypothesized by Van Londen et al., the ΔmGFR_dopa_ may be a measure of the hemodynamic response range of the kidney [19]. It could be that loss of PTC/tubule goes hand-in-hand with overall decreased tubulovascular health in the kidney, resulting in a diminished hemodynamic response to dopamine infusion, but more detailed data on renal hemodynamics are needed to further substantiate this. This would be in line with the hypothesis by Johnson et al. that subtle tubulointerstitial injury with PTC rarefaction makes individuals (and experimental animals) prone to develop salt-sensitive hypertension [33, 34]. Contrary to PTC/tubule, glomerular volume was not associated with pre-donation ΔmGFR_dopa_. It is known that glomerular enlargement is accompanied by an increase in single-nephron GFR [1, 2], which is also demonstrated in our study by a positive association between glomerular volume and pre-donation mGFR. We expected that an increase in glomerular volume would result in smaller ΔmGFR_dopa_, but this was not seen in our cohort. Power could be an issue here or maybe this association does not exist in a healthy population. In multivariable analysis, glomerular volume and PTC/tubule had an additive effect on ΔmGFR_dopa_, suggesting that their effects are partially independent. It might be that in individuals with larger glomerular volume, PTC/tubule provides information on the efficacy of the tubuloglomerular feedback mechanism after “renal stress”.

It has been thought that glomerular enlargement, i.e. hypertrophy, is a compensatory mechanism in response to an increased metabolic or hemodynamic demand and that over time it could lead to glomerulosclerosis, proteinuria and kidney function decline [35–37]. Consistent with this theory and in line with previous literature, the current study shows a positive and significant association of glomerular volume with blood pressure, waist/hip-ratio and BSA and borderline significant with BMI, all established risk factors of CKD (i.e., nephron loss) [38–40]. In a large U.S. cohort, glomerular volume is associated with a post-donation mGFR < 60 mL/min/1.73m^2^ [20], and with a ten-year post-donation mGFR < 45 mL/min/1.73m^2^ (but not < 60 mL/min/1.73m^2^) [12]. However, our study showed that larger glomerular volume was positively associated with three-month- and five-year post-donation mGFR. When comparing the characteristics of our donors to the aforementioned studies, the contrary results could possibly (partly) be explained by the seemingly higher BMI and lower pre-donation eGFR in the U.S. cohort (Mayo Clinic) compared to our cohort, which are both risk factors for lower post-donation kidney function [12]. In addition, glomerular density seemed higher in our cohort, compared to the U.S [14, 41]. Possibly, there was a lower number of nephrons in individuals in the U.S. cohort, whereas in our cohort glomerular enlargement may have remained within physiological ranges. Physiological enlargement of glomeruli is supported by Lenihan et al. who postulated that glomerular hypertrophy post-donation is probably attributable to an increase in the glomerular ultrafiltration coefficient (*K*_f_) and not to glomerular hypertension [5]. Moreover, recent findings in our cohort showed that a stronger short-term increase in post-donation single-kidney GFR, possibly accompanied by glomerular enlargement, predicted better five- and ten-year post-donation GFR [42]. Another reason for the contradictory results could be that kidney function impairment resulting from glomerular hypertrophy was not captured by the follow-up time in our cohort. More studies with greater sample size and follow-up beyond five years are warranted to clarify these discrepancies.

Strengths of this study include the precise kidney function measurements, and the presence of dopamine-related renal function. Furthermore, our study is the first to study PTC density in relation to glomerular morphology and kidney function in healthy individuals. Although we did a power calculation, our study consisted of a small sample size, increasing the risk of missing effects due to limited power. Secondly, we used biopsies taken from living donors before surgery, during surgery and/or after surgery (respectively T1, T2 and/or T3 biopsies). We cannot exclude that the surgical procedure affects PTC density, although in living donors with only little ischemic damage this effect is deemed small [3]. Furthermore, biopsies of different regions of the kidney may have been taken; however, Denic et al. found that clinical characteristics show similar associations with glomerulosclerosis and glomerular volume at different cortical depths [36]. In addition, we found similar associations of glomerular morphology with clinical characteristics as previous studies, supporting the validity of our biopsies. Finally, the majority of our donors were Caucasian, making conclusions not generalizable to other ethnicities.

In conclusion, we found no association of PTC density with clinical characteristics or pre- and post-donation-measured GFR, while glomerular volume is associated with pre-donation blood pressure, body size measurements and GFR. Measurement of PTC density may not provide prognostic information on kidney function after living kidney donation. Our findings support that glomerular and tubular enlargement in healthy kidneys may not be accompanied by an increase in peritubular capillaries. The association of the ratio between peritubular capillaries and tubules with kidney function after dopamine infusion may provide information on hemodynamic response mechanisms and warrants further investigation. Lastly, the relationship between peritubular capillaries and glomerular and tubular parameters in the preservation of renal function merits further study in health and disease.

## Data Availability

The datasets generated during and/or analyzed during the current study are not publicly available due to privacy of the research participants but are available from the corresponding author on reasonable request.
